# Outbreaks in care homes may lead to substantial disease burden if not mitigated

**DOI:** 10.1098/rstb.2020.0269

**Published:** 2021-07-19

**Authors:** Ian Hall, Hugo Lewkowicz, Luke Webb, Thomas House, Lorenzo Pellis, James Sedgwick, Nick Gent, on behalf of the University of Manchester COVID-19 Modelling Group and the Public Health England Modelling Team

**Affiliations:** ^1^ Department of Mathematics, University of Manchester, Manchester, UK; ^2^ The Alan Turing Institute, London, UK; ^3^ IBM Research, Hartree Centre, SciTech Daresbury, Warrington, UK; ^4^ Public Health England, Field Service, London, UK; ^5^ Public Health England, Emergency Response, London, UK

**Keywords:** care home, institutional outbreaks, mathematical modelling

## Abstract

The number of COVID-19 outbreaks reported in UK care homes rose rapidly in early March of 2020. Owing to the increased co-morbidities and therefore worse COVID-19 outcomes for care home residents, it is important that we understand this increase and its future implications. We demonstrate the use of an SIS model where each nursing home is an infective unit capable of either being susceptible to an outbreak (S) or in an active outbreak (I). We use a generalized additive model to approximate the trend in growth rate of outbreaks in care homes and find the fit to be improved in a model where the growth rate is proportional to the number of current care home outbreaks compared with a model with a constant growth rate. Using parameters found from the outbreak-dependent growth rate, we predict a 73% prevalence of outbreaks in UK care homes without intervention as a reasonable worst-case planning assumption.

This article is part of the theme issue ‘Modelling that shaped the early COVID-19 pandemic response in the UK’.

## 1. Introduction

Institutional communities have the potential, upon establishment of a respiratory disease, to suffer a large proportion of the population within becoming infected [1]. Early reports of a novel infection circulated in December 2019 and in January 2020 this infection was attributed to a novel coronavirus, SARS-CoV-2. Since then, the disease has become a pandemic with documented outbreaks in most countries [2].

Outbreaks have been reported in many settings. The outbreak of COVID-19 on board the cruise ship Diamond Princess first came to attention when Japanese authorities quarantined the vessel in Yokohama on 5 February 2020 [3]. Care homes conceptually share similarities to cruise ships in terms of having a stable resident population and a staff population (the period of stability of the population here is similar to or longer than the duration of an outbreak). However, in a care home context the staff population is much better connected to a wider community and the frailty of the residential population is greater, meaning that contact rates are likely higher and eventual outcome worse. Internationally there have been a number of outbreaks in care homes, with substantial associated attack rates and mortality [4]. Analysis of outbreaks of influenza in institutional (enclosed) communities has shown that, while the particular kind of society (prison, care home, school, barracks, etc.) was not a significant explanatory variable of attack ratio, a person’s occupation within the society was [5].

In this analysis, we define a care home in England as premises that the Care Quality Commission (CQC, [6]) monitors, inspects and regulates and in which clients live permanently (or in some situations temporarily owing to, say, a hospital discharge package). Residential settings include care homes for older people, people with learning disabilities, looked-after children, people with mental health problems or substance misuse problems, and hospices. A care home may or may not then have nursing care and so the vulnerability of the residents within will vary from home to home, so it is difficult to generalize a representative care home.

Care homes are at risk of disease importation through connections with community (via staff and visitors) and hospital settings (the frailty of resident population means trips to hospital increased and further discharges to care homes were arranged to ensure capacity on hospital wards). In this study, we consider data from Public Health England (PHE) on care homes reporting outbreaks, applying statistical techniques to the data to forecast eventual potential burden.

## 2. Methods

### (a) Data

**Figure 1.**
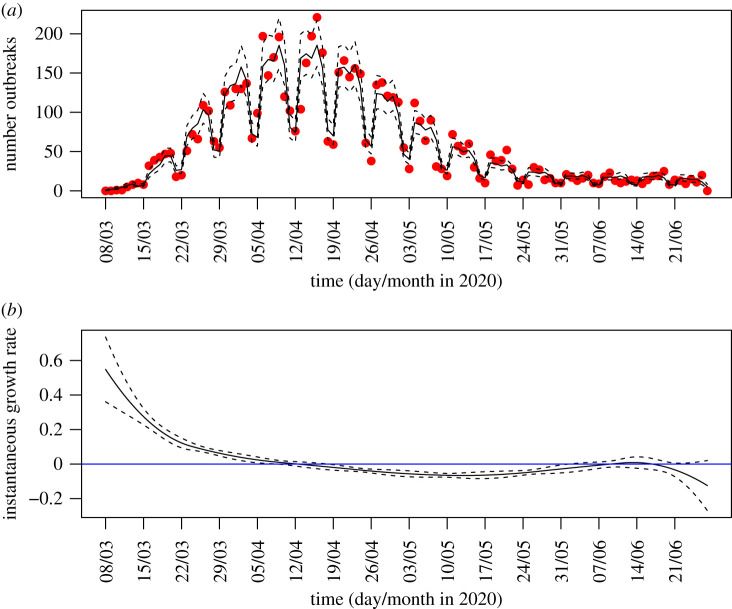
(*a*) Number of care home outbreaks reported each day to Public Health England [7] (red dots), generalized additive model (GAM) best-fit curve (black line) and CI (dashed lines) (quasi-Poisson link with spline on time and fixed effect on weekend or weekday). (*b*) Instantaneous growth rate in number of reported care homes in England derived from spline generated by GAM (solid black line), and 95% confidence interval on growth rate (dashed lines); horizontal blue line shows zero growth for context. (Online version in colour.)

The data, reported to PHE by its network of Health Protection Teams, showed the number of care homes (i.e. homes registered with CQC providing residential or nursing care) newly reporting an outbreak of at least one case each day in England, for a 16 week period from 8 March 2020 to 27 June 2020, and is visualized in figure 1. An updated version is published online weekly by PHE [7].

Further data were available with the postcodes of the affected care homes so we can match this to the CQC register of care homes to consider the risk of outbreaks over space. Where multiple care homes are registered in a single postcode these homes were aggregated to find, say, the reported number of care home beds in that postcode area.

Some care homes report the outbreak status within the home *at the time of report* (number of cases etc.) but there is no mechanism for reliably updating this information at present so a dynamical model within care homes is of limited benefit based on these data.

### (b) Statistical analysis

A generalized additive model (GAM) was used to look at trends over time (in R using mgcv with negative binomial family) [8] in England. Dips in reporting at weekends can be seen and a day of week term was included as an additional fixed effect explanatory variable alongside day of report, which is fitted with a P-spline (chosen over other smoother options to control for wiggliness).

As a separate analysis, the PHE outbreak data may be linked to the CQC register [6] at postcode spatial scale to create a dataset of presence (those homes with an outbreak) and absence (those without an outbreak) to a specific time point. A Gaussian process smoother is used for the spatial term (using Easting and Northing of the postcode centroid converted to kilometre scale), with a thin-plate spline for care home size, and an indicator for urban rural split as additional explanatory variable. This spatial analysis is modelled with a binomial family GAM (implemented in mgcv as above).

The Gaussian process smoother was tested with both a power exponential with exponent equal to one and a Matérn correlation function with *κ* = 1.5 and a value of *ρ* chosen that provided the lowest restricted maximum-likelihood score (REML) [8].

### (c) Mathematical modelling

Care homes are in two states, either showing no cases of disease or with some cases detected. Given the number of care homes *N* is fixed we can consider just the number of care homes reporting at least one case, rather than the proportion.

The number of care homes with at least one case, *I*, will increase by further cases being detected in previously unaffected care homes. It will decrease when laboratory confirmation arrives that the infected cases are not COVID-19 (fast timescale of a couple of days) or when the care home outbreak is declared over. Once declared infection-free a care home may be reinfected later in the pandemic.

Assuming an average duration *T* can be constructed between the fast-acting timescale of laboratory confirmation and slower timescale of outbreak cessation, then *I* → *I* − 1 at some rate *γI*, where *γ* = 1/*T*.

The increase in *I* is harder to model accurately. We assume there is a between-care-home force of infection at rate Λ so that *I* → *I* + 1 with rate Λ(N−I), where *N* is the number of care homes in total.

A key question is what is an appropriate form of Λ. It is well established that an SIS (susceptible–infected–susceptible) model will attain a steady state [9] with constant parameters, but we consider a variety of possible forms of Λ.

If the natural transmission rate is unaffected by future interventions (and so the time variation in force of infection ‘locks’ onto a steady state), then we can project from the plateau to estimate a reasonable worst-case attack rate. The eventual prevalence of care homes suffering an outbreak if this trend continued which would be *P*_∞_ = *TQ*_∞_/*N*, where *T* is the recovery time, *Q*_∞_ is the number of the care homes in England that report each day at steady state incidence and *N* is the total number of care homes in England. According to the CQC, the number of care homes is *N* = 15 517 as of April 2020 [6].

Alternatively, ‘recovered’ care homes can be removed from the data (unless homes have experienced major outbreaks they will remain susceptible to reintroduction of disease) and so we may consider the first reported outbreak in each care home. In this case *γ* → 0 and we define the infection rate to be Λ(t)=β(t)I/N∼O(1). The consequence of this is that

$$I(t)=\frac{N}{1+\exp(-B(t))},$$

where $B(t)=\int_{0}^{t}\beta (t)\;dt$. This is the form of the canonical link function of a binomial family within a generalized linear modelling framework. Indeed, in the case of the additive model above, the function *B* is represented by a constant term and a spline $\mu_{0}+s(t)=\int_{0}^{t}\beta (t)\;dt$ and so the infection rate $\beta (t)=\dot{s}(t)$. Note that if *β*(*t*) = *β*, a constant, then we recover the form expected when the force of infection is proportional to the number of infected care homes.

This latter formulation would provide a very simple framework for estimating *β*(*t*), but the time series is not independent of previous observations (the number of infections on a given day is related to number of infections in the past). While the central estimate may be representative of the trend, the uncertainty will not be well characterized, which will require further investigation.

## 3. Results

Figure 1 shows the number of new reported outbreaks of COVID-19 in care homes in England, which rose rapidly in early to mid-March. The early rapid growth in new reported outbreaks is consistent with rapid growth observed in other surveillance schemes, which was a major cause of concern [10]. In late March, the number reported plateaued for three weeks before dropping in late April to level off in June at relatively small numbers of outbreaks being reported.

There is a clear weekend lull in reporting outbreaks. Table 1 shows the scaling factors for each day of the week using sixteen weeks and eight weeks of data. Saturday and Sunday have the lowest numbers of outbreaks reported, with around 50% fewer than on a Monday; other days have similar reporting rates to Monday. There is little relative difference between week day effects based on eight weeks and sixteen weeks of data.

**Table 1.** The (non-spline) model coefficients and associated standard errors (in brackets) for each day of the week in the temporal model.

day of week	16 weeks	8 weeks
Monday	3.75 (0.067)	4.21 (0.079)
Tuesday	3.79 (0.066)	4.25 (0.078)
Wednesday	3.76 (0.066)	4.24 (0.077)
Thursday	3.86 (0.065)	4.35 (0.075)
Friday	3.71 (0.066)	4.21 (0.076)
Saturday	3.02 (0.076)	3.53 (0.085)
Sunday	2.93 (0.078)	3.4 (0.09)

**Figure 2.**
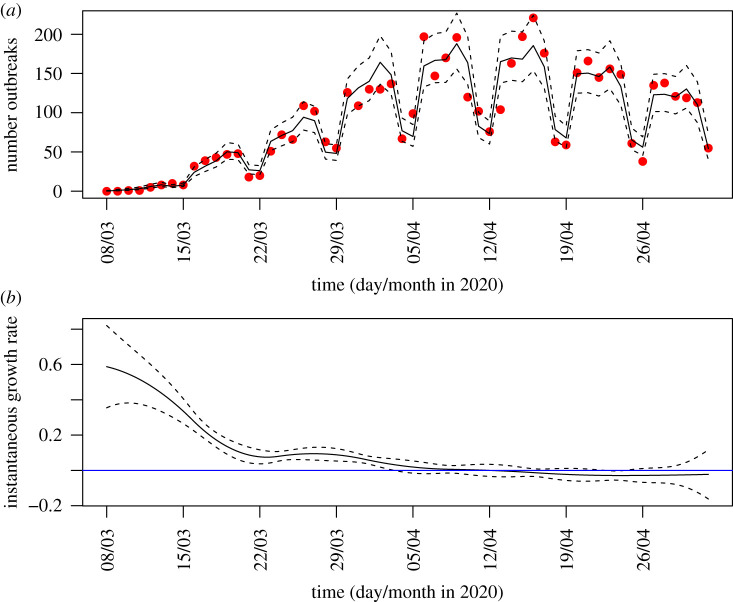
(*a*) Number of care home outbreaks reported each day to Public Health England [7] (red dots), generalized additive model (GAM) best-fit curve (solid black line) and CI (dashed lines) (quasi-Poisson link with spline on time and fixed effect on weekend or weekday). (*b*) Instantaneous growth rate in number of reported care homes in England derived from spline generated by GAM (solid black line), and 95% confidence interval on growth rate (dashed lines); horizontal blue line shows zero growth for context. (Online version in colour.)

Figures 1*b* and 2*b* show the derivative of the spline arising from the GAM. If this derivative is constant and positive then this is a sign of exponential growth, if constant and negative, of exponential decay, and if it is tracking the horizontal blue line (a reference line for zero growth) this is suggestive that the data is plateauing. There is some evidence from the upper 95% confidence interval touching zero of overall downward trend in late April as well as the more recent evidence of plateau in June.

Figure 2 shows the data for an eight week period up to the start of May. Considering this censored time series it can be seen that the data during April seemed to be in a state of plateau. The SIS theory predicts a steady state of a constant fraction of new care homes reporting an outbreak and so the observed trend can be projected into the future. These local hot-spots of outbreaks are suggestive of local patterns of transmission and connectedness of local care homes rather than a random series of outbreaks.

We find an upper limit estimate on *Q*_∞_ = 190/*N* from the two week plateau seen in the data and fit by the GAM, which we use as a worst-case scenario. The recovery time of a care home is uncertain. If the generation time is taken to be 5 days [11] and 3–4 generations of disease occur with associated wait of 14 days after last observed case, then *T* ≈ 34 is plausible, meaning that *P*_∞_ = 0.41. If *T* = 60 days (allowing for weaker infection control and undiagnosed cases) then *P*_∞_ = 0.73 is potential reasonable worst-case planning end state.

**Figure 3.**
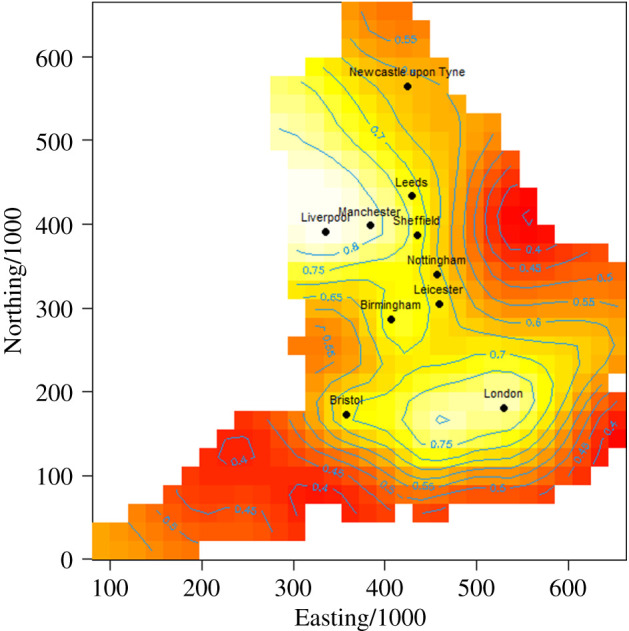
Proportion of care homes reporting an outbreak to PHE up to 1 May 2020 (comparable to the data in figure 2). Spatial smoothing is performed with a binomial family generalized additive model with Gaussian process smoothing on Easting and Northing (of postcode centroid) and thin-plate spline on care home size (based on CQC registration), with fixed effect of urban/rural categorical variable.

Figure 3 shows the results with the Gaussian process smoother used over space. The Matérn function shown has the smallest REML, with *ρ* = 58. This approach identifies the increase risk of outbreaks occurring in the North West of England focused on Liverpool, as well as other hot-spots such as Oxfordshire and London.

**Figure 4.**
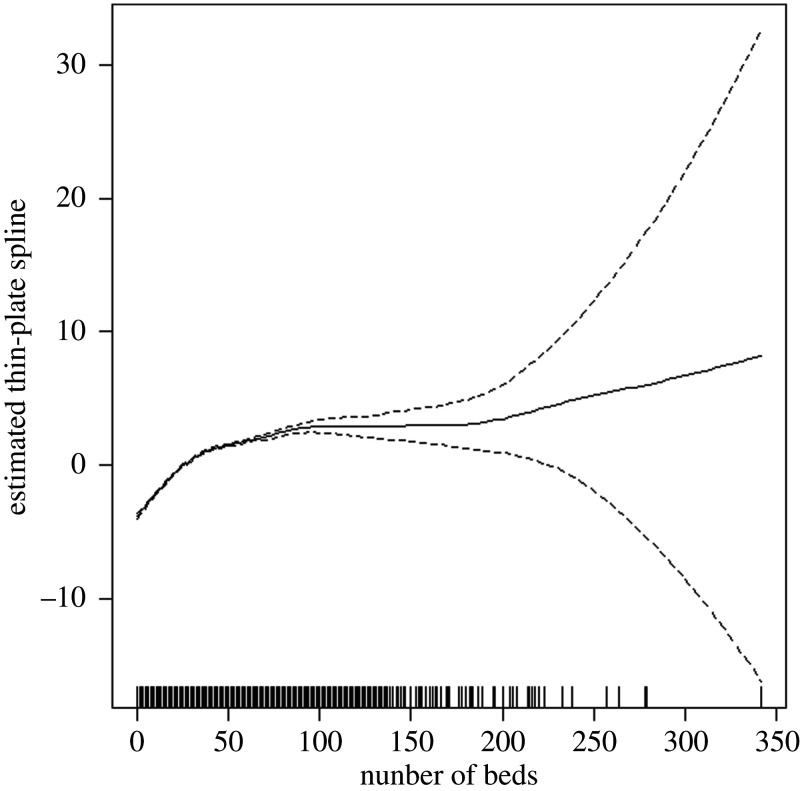
Estimated thin-plate spline obtained from the spatial GAM based on the number of beds registered with CQC in a care home based on eight weeks of data.

Figure 4 shows the fitted spline for the number of beds shown in the CQC register. This suggests a fairly linear increase in risk for care homes of declared size below 30 beds before the risk levels off (though becoming more uncertain) as number of beds within a home increases. The model intercept has a value of 0.174 (s.e. 0.072), while postcode with care homes that are classed as Urban have an odds ratio of 1.166 (0.999, 1.36).

## 4. Discussion

This analysis is relatively simple and does not address the size of the outbreaks within homes themselves. This will be variable based on local outbreak management and reporting. Bringing the space and time models together in a single framework is ongoing work. However, it is important to observe that the 73% value is the prevalence of care homes with outbreaks and not the incidence within. This means homes should expect to suffer multiple importations over time until local herd immunity is reached, provided there is no change in behaviour or bio-security policy affecting ingress. These outbreaks will accumulate cases so the cumulative attack rate within a home may be large owing to a mix of explosive outbreaks and repeated importation.

The early growth in the data that reduces the ability of fitting a constant force of infection may be an artefact of surveillance (a relatively new scheme having improved uptake in usage over time) rather than disease-driven. Additionally, the importation of infection may be higher in early stages (i.e. prior to ‘lockdown’) from the community (i.e. visitors) or from cases being discharged from hospital. In England, some interventions were implemented on 24 March 2020, when enhanced testing of hospital transfers and cessation of visitors may have started to reduce opportunities for disease ingress to care home settings, but recommendation of these mitigation strategies evolved over time; for example, it was not until 14 June that care homes were advised to quarantine all new residents on admission. Changes in policy do not universally lead to changes in practices during the pandemic.

A plausible explanation of the model presented here is that the vehicle connecting care homes is the staff. Staff seem to be suffering disease at similar numbers to residents where it is reported to PHE (it should be noted that the reason for staff absence is unclear in the data and it may be that staff are absent for precautionary reasons or care of dependents elsewhere, rather than owing to the disease directly). If staff work in multiple care homes then these high attack rates may lead to depletion of susceptible staff and so reduce transmission rates over time. However, recent statements from WHO suggest evidence for long-lasting immunity is limited [12]. Moreover, staff interact with their households and the community and other care homes, so infection can be passed between care homes with staff acting as the main vectors. This is not a sign that staff are being unobservant of the current severity of the situation but highlights the challenges they face and the high numbers of cases with mild symptoms.

More recent data up to 23 July 2020 have shown a reduction in outbreaks being reported [7]. This trend is a positive sign and may be a sign of changing policy in some areas. This suggests the reasonable worst-case scenario of 73% of care homes being in outbreak status at any given time is not going to be observed, but it should be noted that this modelling was originally conducted in mid-April at the peak of outbreaks being reported and changes in practice rapidly introduced as a result of the original assessment may have had a role in the reduction. Further work will be conducted to bring together the spatio-temporal statistical modelling with more detailed epidemic modelling.

Further investigation is imperative for understanding the outbreaks within care homes, explicitly including the role of staff. This can be established through enhanced testing and monitoring the interactions of care homes with the wider community. It also suggests the need for support intervention to assist outbreak management and hard-pressed staff working in the social care sector.

## In context

The Covid-19 pandemic placed significant pressure not only on UK's hospitals, but also on the resources and capacity of the non-hospital healthcare system. This pressure was particularly acute in social care settings, which have a triple risk of (1) a closed population that are (2) highly vulnerable to infection and (3) transmission is boosted due to close and frequent contact. Indeed, both residential and nursing care homes experienced significant outbreaks of Covid-19 and high mortality rates. In England, there are approximately 15 000 care homes with 450 000 beds.

On 9 March 2020, UoM researchers submitted a paper to SPI-M that quantified the likely impact of unconstrained Covid-19 spread in care homes, in terms of hospitalisations and deaths (results from which were later presented in Overton *et al*. [13]).

This SPI-M report led directly to conversations with colleagues in Department of Health and Social Care (and HMPPS) about reasonable worst case scenarios for closed societies (during w/c 30th March) which provided access to reported outbreaks in care home settings from 9th April onwards. On the 19th April initial analysis was shared with SPI-M based on projections using a Generalised Additive Model and ‘SIS’ model approach that forms the basis of this special issue. This report forecast 90% of care homes would be in a state of outbreak at the same time if the existing trend continued. On the 11th May the medRxiv submission was made with a slightly lower estimate of attack rate in care homes based on longer time-series of data.

The week after the first SPIM report (24th April) the first care home analysis group meeting was held, and a week later the group formally became the SAGE working group on care homes. This group then submitted a paper to SAGE on 12 May 2020 [14] which contained further modelling analysis (from range of modelling groups) looking at testing strategies and evidence that made the case for collecting better-quality data and testing of both residents and staff within care homes. The number of outbreaks has subsequently changed over time due to combination of national lockdown's reducing force of infection into care homes, the role of testing staff and residents and specific infection control within care home settings which is currently subject to ongoing evaluation and review.

## Data accessibility

A publicly available version of the data is cited; full release of data is not possible owing to the the disclosure status of data.
